# One-Step Fabrication of Poly(vinylidene Fluoride-Co-Hexafluoropropylene)/Perfluorodecyltriethoxysilane Fibrous Membranes with Waterproof, Breathable, and Radiative Cooling Properties

**DOI:** 10.3390/molecules30040763

**Published:** 2025-02-07

**Authors:** Aohan Hou, Juan Xie, Xiaohui Wu, Guichun Lin, Yayi Yuan, Xi Liu, Yancheng Wu, Feng Gan, Yangling Li, Yuxiao Wu, Gang Huang, Zhengrong Li, Jing Zhao

**Affiliations:** 1 College of Textile Science and Engineering, Wuyi University, Jiangmen 529020, Chinawuyuxiao12389@126.com (Y.W.);; 2 School of Textile and Garment, Anhui Polytechnic University, Wuhu 241000, China

**Keywords:** one-step electrospinning, waterproof and breathable, radiative cooling

## Abstract

Functional membranes with waterproof, breathable, and thermal regulation capabilities are increasingly sought after across various industries. However, developing such functional membranes commonly involves complex multi-step preparation processes. Herein, we introduced perfluorodecyltriethoxysilane (FAS) into the poly(vinylidene fluoride-co-hexafluoropropylene) (PVDF-HFP) solution for one-step electrospinning, successfully fabricating membranes that combine these properties. The hydrophobicity of the PVDF-HFP/FAS membrane was greatly improved with the water contact angle increased from 120.6° to 142.9° and the solar reflectance rising from 72% to 92% due to the presence of fluorocarbon segments. The synergistic effect of enhanced hydrophobicity, small pore size, and elevated solar reflectivity resulted in robust water resistance (62 kPa), excellent water vapor transmission rate (12.4 kg m^−2^ d^−1^), and superior cooling performance (6.4 °C lower than commercial cotton fabrics). These findings suggest that the proposed PVDF-HFP/FAS membranes, characterized by desired multifunction characteristics and scalable production, hold great potential for application in diverse strategic fields.

## 1. Introduction

In recent years, functional textiles have garnered significant attention for their dual role as protective barriers and as providers of comfort, durability, and specialized properties [1,2]. Among these, waterproof and breathable membranes (WBMs) stand out as advanced functional textiles capable of preventing the penetration of liquids and pollutants while effectively transferring sweat vapor [3,4,5]. As a result, WBMs are widely used in various applications, including construction materials, protective clothing, outdoor gear, and electronic devices. These membranes generally fall into two types: hydrophilic nonporous membranes and hydrophobic microporous membranes. Due to their extensive network of interconnected micropores, hydrophobic microporous membranes offer superior comfort compared to their nonporous counterparts [6,7,8].

At present, several approaches have been developed to fabricate hydrophobic microporous membranes, such as phase separation, mechanical stretching, and template-based techniques [9,10,11]. However, these conventional approaches often face limitations, including restricted material options, inadequate thermal-moisture comfort, and challenges in simultaneously regulating pore size and porosity. Therefore, developing WBMs that combine high waterproofness, breathability, and thermal regulation through a simple and scalable process remains a significant challenge.

Electrospinning, a pioneering technique for nanofiber production, has emerged as a promising solution for fabricating nanofibrous membranes with fine fiber diameters, tunable pore sizes, and variable wettability [12,13,14]. This technology has facilitated the creation of hydrophobic microporous membranes from a variety of polymers, such as cellulose acetate, polyamide 6, polyacrylonitrile, polyvinylidene fluoride (PVDF), and polyurethane [15,16,17,18,19]. According to previous studies, the waterproof, breathable properties of electrospun fibrous membranes are primarily derived from their interconnected pore structure and hydrophobic surfaces [20,21]. The construction of a hydrophobic surface can be achieved either by directly electrospinning hydrophobic polymers or by modifying hydrophilic membranes to impart hydrophobicity. The former approach is particularly advantageous due to its simplicity and efficiency.

Poly(vinylidene fluoride-co-hexafluoropropylene) (PVDF-HFP), as a copolymer of PVDF, possesses excellent hydrophobicity due to the incorporation of amorphous fluoropropylene segments into the vinylidene fluoride chains [22,23,24,25]. Despite extensive research focusing on the radiative cooling properties of PVDF-HFP, most studies rely on complex, multi-step fabrication processes with high associated costs. To date, little attention has been given to exploring its waterproof and breathable properties, representing a notable gap in the literature.

In the present work, we report a straightforward one-step electrospinning for developing fibrous composite membranes with waterproof, breathable, and radiative cooling properties. PVDF-HFP was chosen as the base polymer for its inherent hydrophobicity, high infrared emissivity, and strong mechanical properties. To further enhance the hydrophobicity and solar reflectance, 1H,1H,2H,2H-perfluorodecyltriethoxysilane (FAS-17, referred to as FAS) was incorporated into the PVDF-HFP solution. A thorough investigation was conducted to assess the effects of FAS on the membrane’s morphology, pore structure, waterproof and breathable performance, and spectral properties. The resulting PVDF-HFP/FAS composite membrane exhibited small pores and high porosity, providing excellent waterproofness, breathability, and cooling performance. These multifunctional membranes show great potential for use in applications such as architecture, outdoor gear, and protective clothing.

## 2. Results

### 2.1. Design of PVDF-HFP/FAS Composite Fibrous Membranes

The design of the multifunctional membrane aimed to achieve waterproof, breathable, and radiative cooling properties based on three key principles: (i) optimal waterproofness and breathability require the membrane to possess hydrophobic channels with small pore sizes and high porosity [26,27]; (ii) effective thermal regulation is achieved by membranes with selective spectral responses that strongly emit human infrared radiation while reflecting solar spectra [28,29,30]; and (iii) the preparation process should be simple and scalable for broader applications.

PVDF-HFP was selected as the base polymer due to its low surface energy, which aids in the formation of hydrophobic surfaces, and its strong infrared emissivity, attributed to the presence of functional C-F bonds [31,32]. FAS was incorporated to further enhance the membrane’s hydrophobicity and solar reflectance, owing to its long-chain perfluoroalkyl groups, which impart water repellency to the fiber surface. The addition of C-F and Si-O-C bonds further contributed to improved solar reflection and infrared emissivity [33,34,35]. By adjusting the properties of the spinning solution and electrospinning parameters, a membrane with a small pore size and high porosity can be obtained.

**Figure 1:**
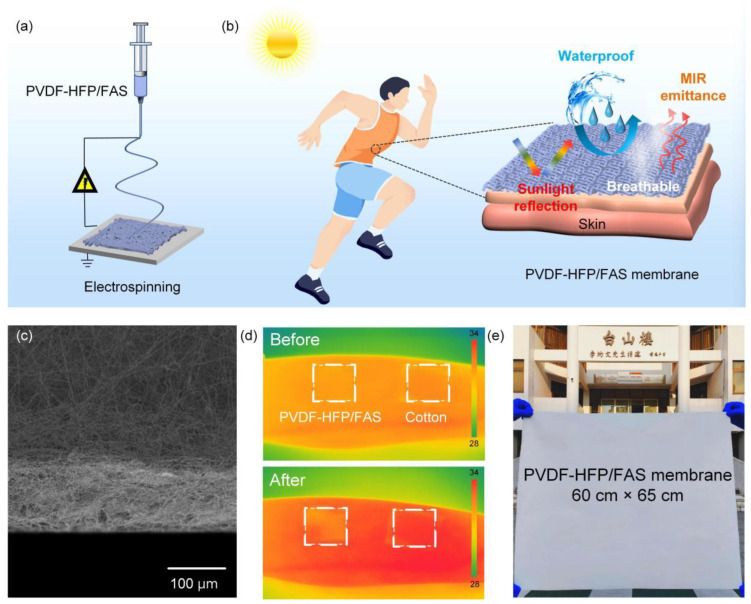
(**a**) Schematic diagram of PVDF-HFP/FAS multifunctional membrane. (**b**) Schematic exhibiting waterproofness, breathability, and radiative cooling of the designed materials. (**c**) Cross-sectional image of the PVDF-HFP/FAS membrane. (**d**) IR pictures of PVDF-HFP/FAS membrane and cotton after 10 min of exposure to sunlight. (**e**) Optical image of the large-sized membrane.

As shown in Figure 1a, the PVDF-HFP/FAS membrane was fabricated using a simple and efficient one-step electrospinning technique. The resulting membrane not only exhibits high solar reflectance and infrared emissivity-key factors for achieving radiative cooling but also effectively prevents liquid penetration while allowing moisture to pass through (Figure 1b). The membrane showed a fibrous and fluffy architecture with a thickness of 220 μm (Figure 1c). The cooling performance is further demonstrated in Figure 1d, where infrared thermal images reveal that the PVDF-HFP/FAS membrane has a lower surface temperature (31.5 °C) compared to cotton fabric (33.6 °C) after 10 min of sun exposure. Additionally, the membrane’s large size (60 × 65 cm^2^) was easily scalable using this fabrication method (Figure 1e), highlighting its potential for use in advanced protective garments.

### 2.2. Structural Analysis of PVDF-HFP Fibrous Membranes

**Figure 2:**
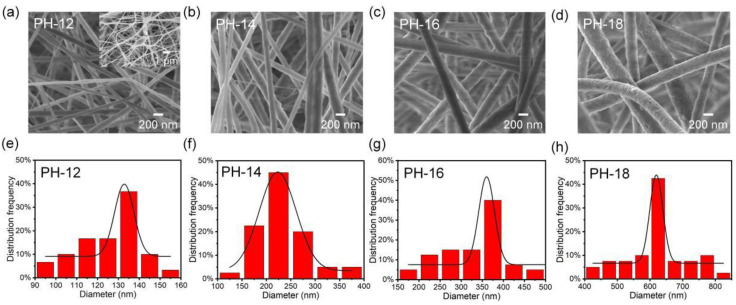
(**a**–**d**) SEM images of different pure PVDF-HFP fibrous membranes. (**e**–**h**) The diameter distributions of corresponding PVDF-HFP fibrous membranes. The number “x” in the PH-x sample refers to the weight percent concentration of PVDF-HFP polymer.

The relationship between the microstructure of the prepared PVDF-HFP fibers and the concentration of the electrospinning solution was investigated, as illustrated in Figure 2a–d. The fibrous membranes prepared with PVDF-HFP concentrations of 12, 14, 16, and 18 wt% are denoted as PH-12, PH-14, PH-16, and PH-18, respectively. The PH-12 fibers exhibited fine diameters with a significant number of beads (see Figure 2a inset), which likely resulted from the low viscosity of the spinning solution [36,37]. Due to this low viscosity, Rayleigh instability prevented sufficient fiber elongation under the applied electric force. As the concentration of the spinning solution increased, the morphology of the PVDF-HFP fibers became more uniform, and the fiber diameters increased accordingly. As shown in Figure 2e–h, the average fiber diameter increased progressively from 133 to 627 nm with increasing concentration. This growth in fiber diameter can be attributed to the rise in solution viscosity (Figure S1), consistent with previously reported findings [18].

**Figure 3:**
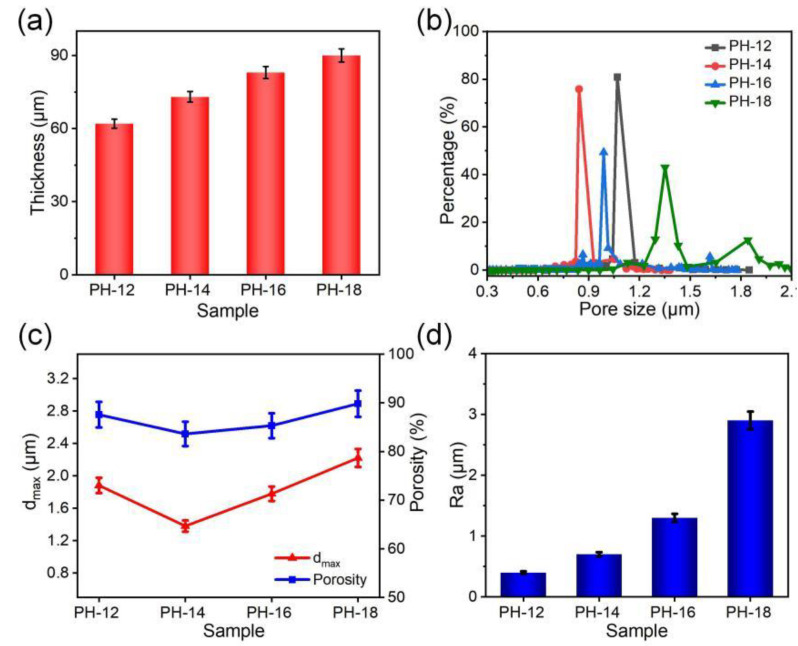
(**a**) Thickness, (**b**) pore size distribution, (**c**) d_max_ and porosity, and (**d**) Ra of different pure PVDF-HFP fibrous membranes.

Moreover, with an increase in concentration of spinning solution from 12 to 18 wt%, the thickness of the PVDF-HFP fibrous membranes increased from 62 to 90 μm (Figure 3a). As depicted in Figure 3b, the average pore size of the PH-12 sample was 1.1 μm, while for the PH-14 sample, it decreased to 0.8 μm due to the reduction in beaded structures. A gradual increase in pore size was observed for the PH-16 and PH-18 samples, likely resulting from the enlargement of fiber diameters. The maximum pore size (d_max_) decreased from 1.8 μm in PH-12 to 1.3 μm in PH-14, corresponding to the fewer beaded fibers, but increased to 2.1 μm in PH-18. The change in porosity is the same as that of pore size, with values varying from 83.6% to 89.8% (Figure 3c).

### 2.3. Properties Analysis of PVDF-HFP Fibrous Membranes

**Figure 4:**
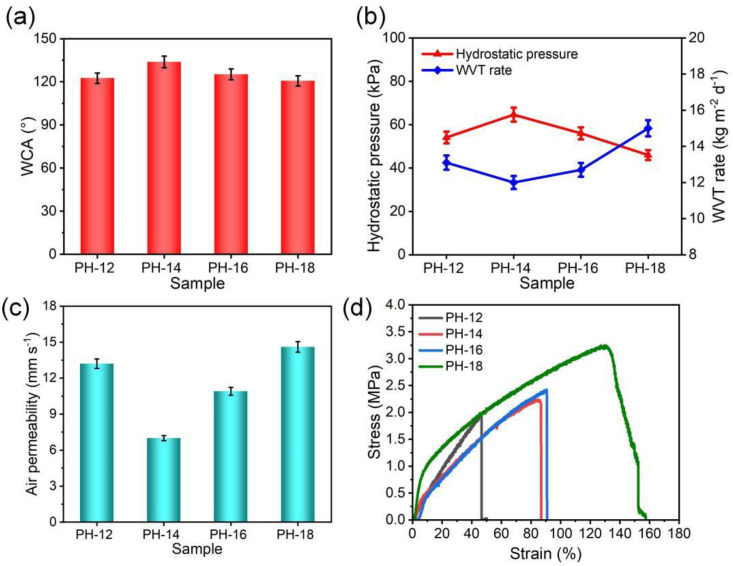
(**a**) WCA, (**b**) water resistance and water vapor permeability, (**c**) air permeability, and (**d**) stress–strain curves of different pure PVDF-HFP fibrous membranes.

Wettability plays a crucial role in determining the waterproof properties of fibrous membranes. Typically, the surface wettability of fibrous materials is influenced by both surface chemical compositions and geometrical microstructure [38,39,40]. As shown in Figure 4a, the static WCA of PVDF-HFP membranes ranged from 122.5° to 120.6°, which could be owing to the similarity in surface chemical composition and surface roughness. In general, the waterproofness of the materials is mainly governed by the pore size and surface hydrophobicity, as described by the Young–Laplace equation [41]:

$$hydrostatic\;pressure=-\frac{4\gamma cos\theta }{d_{max}}$$

where θ is the WCA and γ represents the surface tension of water. Smaller pore size and greater hydrophobicity enhance water resistance. As shown in Figure 4b, from PH-12 to PH-14, the water resistance of the fibrous membrane enhanced from 54.1 to 64.6 kPa, which was derived from the decreased pore size and the denser structure. However, water resistance slightly decreased from PH-14 and PH-18 due to the increased *d_max_* in the fluffier structure. Despite this, the PH-18 sample still retained reasonable waterproof performance at 46 kPa. The breathable mechanism follows Fick’s law [20]:

$$WVT\;rate=-D\frac{dC}{dx}$$

where dC/dx is the concentration gradient, and D is the diffusion coefficient. As polymer concentration increased, the WVT rates were 13.1, 12.0, 12.7, and 15.0 kg m^−2^ d^−1^, correlating positively with porosity (Figure 3c). Additionally, air permeability values for PH-12 to PH-18 were 13.2, 7.0, 10.9, and 14.6 mm s^−1^, respectively, indicating good breathable performance of the fibrous membranes (Figure 4c). The stress–strain curves in Figure 4d show that PH-18 fibers exhibited the highest tensile strength (3.5 MPa) and elongation (127%), likely due to their larger diameters and more homogeneous structures.

**Figure 5:**
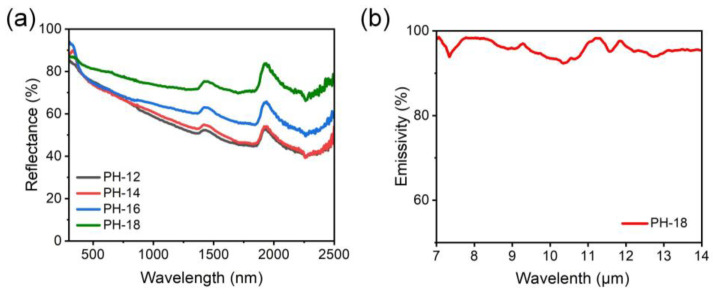
(**a**) Reflectance and (**b**) infrared emissivity of different pure PVDF-HFP fibrous membranes.

Achieving efficient radiative cooling requires high solar reflectance and strong infrared emission [42]. Figure 5a presents the reflectivity of fibrous membranes with varying PVDF-HFP concentrations. The average solar reflectance for PH-12, PH-14, PH-16, and PH-18 membranes was 55%, 57%, 63%, and 72%, respectively (Figure S2). The PH-18 membrane exhibited the highest reflectance, which is likely due to its increased thickness and rough surface morphology (Figure 3d). Furthermore, based on Mie scattering theory, the scattering of light by fibers correlates with fiber diameter [43]. In this work, the fiber diameter distribution of PH-18, ranging between 0.26 and 0.83 μm, lies within the solar spectrum (0.25–2.5 μm), allowing for effective scattering of sunlight and enhanced solar reflectivity.

Besides solar reflectance, infrared emittance is also a critical factor in determining the cooling properties of materials. Figure 5b presents the infrared emittance spectrum of the PH-18 membrane, which showed the highest solar reflectance. The PVDF-HFP fibers exhibited strong thermal radiation emittance and transmittance (εLWIR≈95%) due to the vibrational bonds such as -CH_2_ (1400 cm^−1^), -CF_2_ (1180 cm^−1^), and combined CC/CF_2_ (876 cm^−1^) overlapping with the atmospheric transparency spectral window [44,45]. Given the above discussion, the PH-18 membrane emerges as a promising candidate for effective radiative cooling and for its waterproof, breathable properties. Consequently, it warrants further exploration and detailed examination.

### 2.4. Morphologies of PVDF-HFP/FAS Membranes

**Figure 6:**
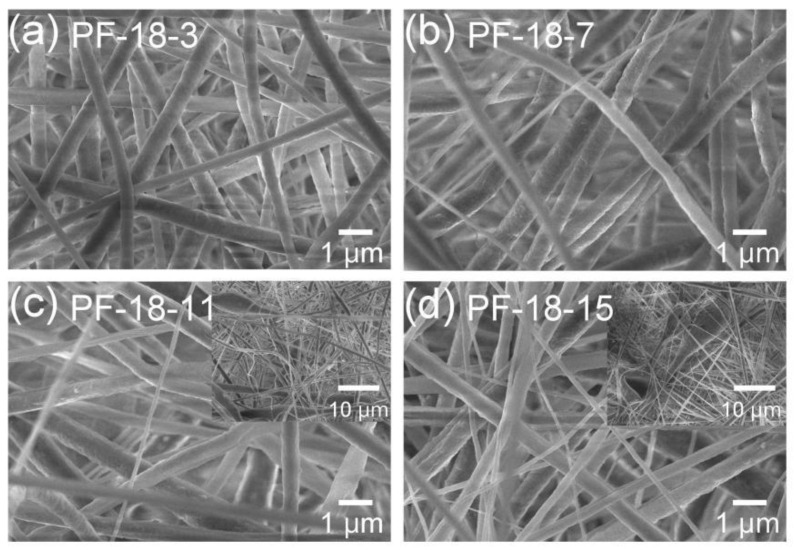
(**a–d**) SEM images of different PVDF-HFP/FAS composite fibrous membranes. The number “y” in the PF-18-y sample refers to the weight percent concentration of FAS.

To further enhance the reflectivity and emissivity of the fiber membrane, thereby improving its radiative cooling effect, FAS was incorporated into the PVDF-HFP fiber membrane. The influence of FAS concentration on the membrane’s morphology and structure was then investigated. The fibrous membranes prepared with PVDF-HFP/FAS solutions containing 18 wt% PVDF-HFP and 3, 7, 11, and 15 wt% FAS are denoted as PF-18-3, PF-18-7, PF-18-11, and PF-18-15, respectively. Figure 6a–d show the morphologies of membranes with varying FAS concentrations. The PF-18-3 sample exhibits relatively uniform fibers with an average diameter of 459 nm. As the FAS concentration increases to 7 wt%, the fiber diameter decreases to an average of 318 nm (PF-18-7). In the PF-18-11 sample, fibers displayed uneven diameters and bead formation, with an average fiber diameter of 293 nm. At the highest concentration, PF-18-15, the presence of beads became more pronounced, and the average fiber diameter further decreased to 118 nm (Figure S3).

### 2.5. Microstructures of PVDF-HFP/FAS Membranes

**Figure 7:**
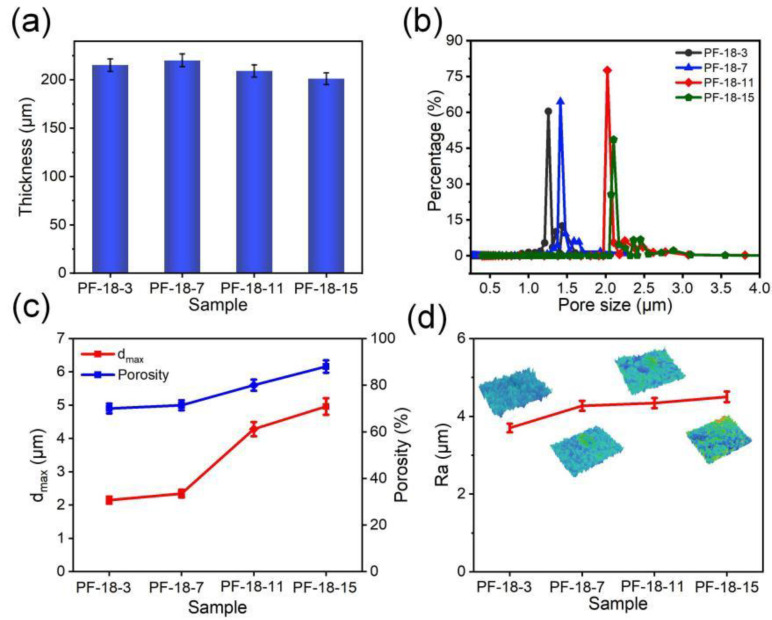
(**a**) Thickness, (**b**) pore size distribution, (**c**) d_max_ and porosity, and (**d**) Ra of different PVDF-HFP/FAS composite fibrous membranes.

As depicted in Figure 7a, the thickness of the fiber membrane initially increased and then decreased as the FAS concentration rose. When the FAS concentration increased from 3 to 7 wt%, the thickness of the PVDF-HFP/FAS membrane remained relatively stable, ranging from 215 μm (PF-18-3) to 220 μm (PF-18-7). The increase in thickness, compared to the pure PVDF-HFP membrane, is primarily attributed to the fluffier fibrous structure. However, at higher FAS concentrations (11 and 15 wt%), the membrane thickness decreased to 209 μm (PF-18-11) and 201 μm (PF-18-15), likely due to enhanced electrostatic repulsion forces between fibers, which negatively affected fiber deposition [46,47,48]. The experiment results in Figure 7b,c further support this explanation, with average pore diameters of 1.3, 1.4, 2.0, and 2.1 μm, and d_max_ of 2.1, 2.3, 4.3, and 4.9 μm, respectively. The porosity of the PVDF-HFP/FAS membranes increased from 70% (PF-18-3) to 88% (PF-18-15), reflecting a decrease in packing density as FAS concentration rose. Additionally, the surface roughness (Ra) increased progressively with FAS concentration, with Ra values of 3.7, 4.2, 4.3, and 4.5 for PF-18-3, PF-18-7, PF-18-11, and PF-18-15, respectively (Figure 7d), which may be associated with the increasing concentration of fluorinated segments [49,50].

### 2.6. Surface Chemistry of PVDF-HFP/FAS Membranes

**Figure 8:**
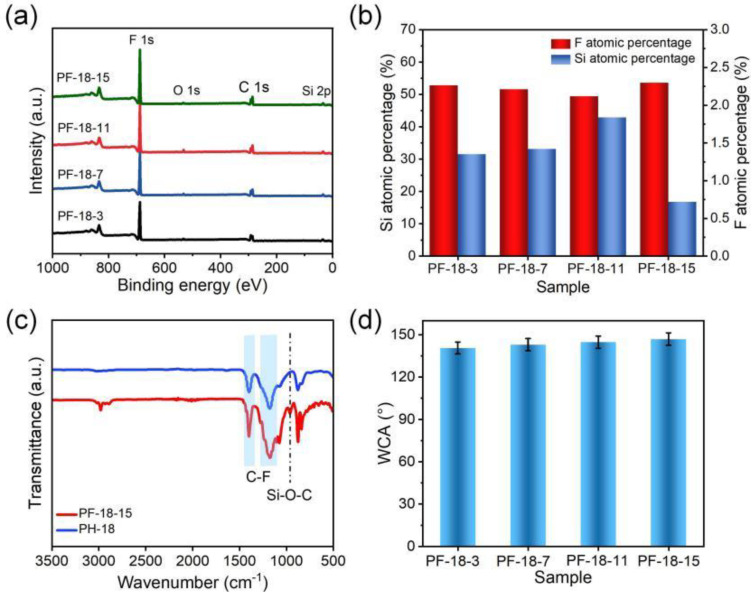
(**a**) XPS, (**b**) Si and F atomic percentage, (**c**) FTIR, and (**d**) WCA of different PVDF-HFP/FAS composite fibrous membranes.

As exhibited in Figure 8a, the XPS spectra for all four membranes displayed consistent characteristic peaks for carbon (297.98 eV for C 1s), oxygen (544.98 eV for O 1s), silicon (109.98 eV for Si 2p), and fluorine (697.98 eV for F 1s) [51,52,53]. As the FAS concentration increased from 3 to 11 wt%, the carbon atom ratio decreased from 48.36% to 45.95%, while the oxygen and silicon atom ratios increased from 1.98% to 2.81% and from 1.35% to 1.84%, respectively. The fluorine atom ratio showed a slight decline, from 52.81% to 49.41% (Table S1). However, when the FAS concentration increased to 15 wt%, the carbon, oxygen, and silicon ratios further decreased to 44.17%, 1.48%, and 0.72%, respectively, while the fluorine atom ratio increased to 53.63% (Figure 8b).

The FTIR spectrum of the PVDF-HFP/FAS composite membrane revealed strong absorption peaks between 1445–1340 cm^−1^ and 1265–1100 cm^−1^, corresponding to C-F stretching vibrations (Figure 8c). Additionally, a peak at 957 cm^−1^ indicated the presence of a Si-O-C stretching band, confirming the incorporation of FAS [54]. As shown in Figure 8d, the WCA increased progressively with FAS concentration, rising from 140.5° to 146.8°, likely due to increased surface roughness.

### 2.7. Properties of PVDF-HFP/FAS Membranes

**Figure 9:**
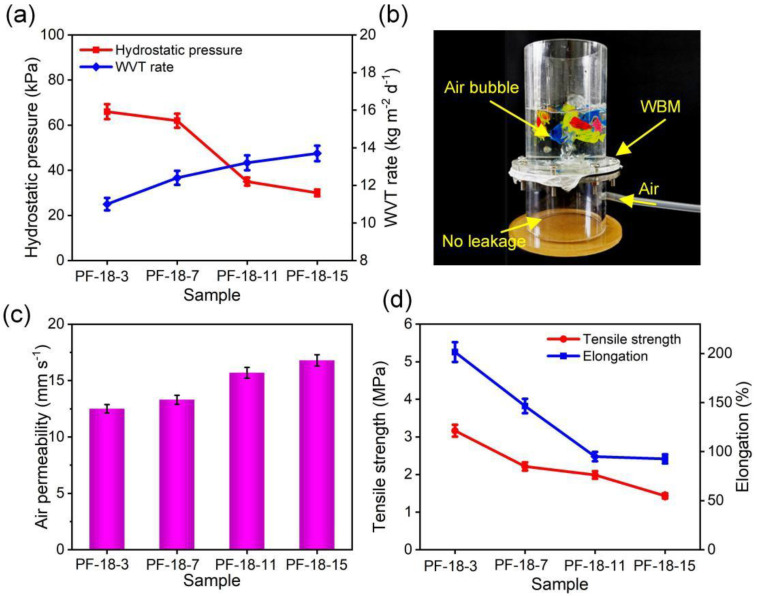
(**a**) Hydrostatic pressure and WVT rate of different PVDF-HFP/FAS composite fibrous membranes. (**b**) Image exhibiting waterproof and breathable performance. (**c**) Air permeability and (**d**) tensile strength and elongation of different PVDF-HFP/FAS composite fibrous membranes.

As exhibited in Figure 9a, the hydrostatic pressure of the PF-18-3, PF-18-7, PF-18-11, and PF-18-15 membranes, containing FAS concentrations of 3, 7, 11, and 15 wt%, were 66, 62, 35, and 33 kPa, respectively, which might be resulted from the enlarged pores. The water vapor permeability of the PVDF-HFP/FAS membranes increased successively from 11.0 to 13.7 kg m^−2^ d^−1^, which can be attributed to the improved porosity. Figure 9b demonstrates the waterproofness and breathability of the membranes, with one piece of membrane resisting the penetration of 2 L of water while still allowing moisture transport.

Additionally, the PVDF-HFP/FAS membranes showed good air permeability, which improved from 12.5 mm/s (PF-18-3) to 16.8 mm/s (PF-18-15) as the FAS concentration increased from 3 to 15 wt% (Figure 9c). However, the tensile strength decreased from 3.2 MPa (PF-18-3) to 1.4 MPa (PF-18-15), and the elongation at break reduced from 202% to 93%, likely due to the formation of beads on the fibers, which negatively impacted the mechanical properties (Figure 9d).

**Figure 10:**
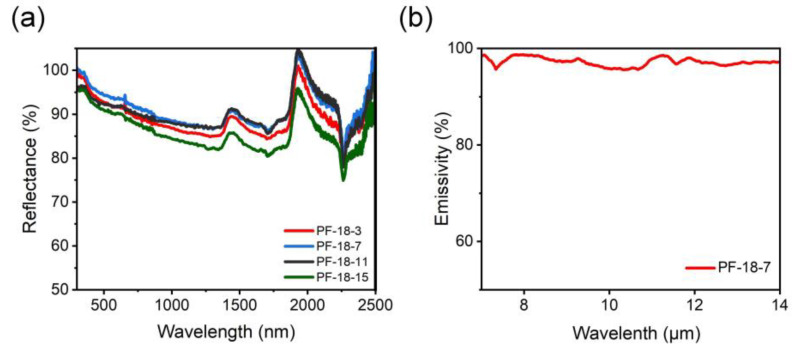
(**a**) Reflectance of different PVDF-HFP/FAS composite fibrous membranes. (**b**) Emissivity of the PF-18-7 sample.

As shown in Figure 10a, the reflectivity of the membranes increased significantly with the introduction of FAS. When the FAS concentration increased from 3 to 7 wt%, the reflectivity of the PVDF-HFP/FAS composite membranes rose from 89% (PF-18-3) to 92% (PF-18-7) (Figure S4). However, the reflectivity of PF-18-11 (90.5%) and PF-18-15 (86%) decreased, which can be attributed to the reduced fiber diameter and thickness of these membranes. Thus, the PF-18-7 sample exhibited the highest solar reflectivity. Additionally, the emissivity of the PF-18-7 sample was measured at 97.2%, which is 2.2% higher than that of the pure membrane (Figure 10b).

**Figure 11:**
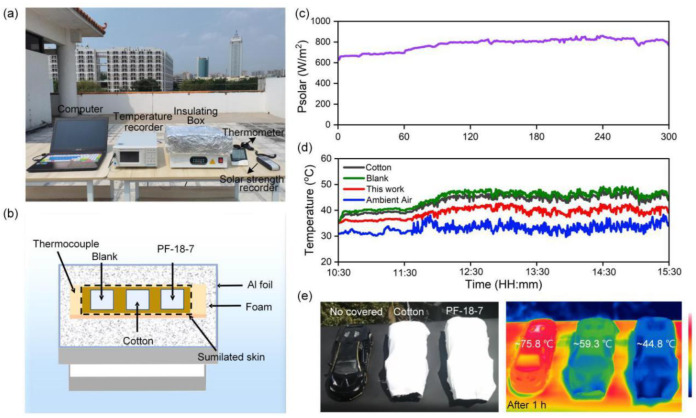
(**a**,**b**) Photograph and schematic of the apparatus used to test the outdoor cooling effect. (**c**) The recorded solar radiation. (**d**) The real-time temperatures of ambient air, uncovered, cotton-covered, and PF-18-7 sample-covered skin simulator. Temperature data recorded on a clear day in Jiangmen, China (27 September 2024 10:30 to 15:30). (**e**) The digital and infrared images of the models without cover, covered with cotton, and covered with PF-18-7 sample.

We investigated the outdoor daytime cooling performance of the PVDF-HFP/FAS membrane at Wuyi University, Jiangmen, China. Figure 11a,b exhibit the optical photos and schematic diagram of the self-designed temperature-measuring device, which was placed directly under sunlight during testing. The temperature of the PF-18-7 sample was tested using a thermocouple, while the temperatures of the control groups (cotton-covered and blank) and ambient air were monitored for comparison. The recorded solar irradiation intensity is depicted in Figure 11c. The temperature of the skin simulator was monitored continuously under clear skies for 5 h. The skin simulator temperature covered with the PF-18-7 sample maintained temperatures 6.4 °C and 10.3 °C lower than those of cotton-covered and uncovered skin simulators, respectively (Figure 11d).

We further examined the cooling capabilities of PVDF-HFP/FAS composite membranes. Three identical toy cars were positioned on a flat surface, with the left car uncovered, the middle car covered with cotton, and the right car covered with PF-18-7 sample (Figure 11e). After 1 h of solar exposure (729 W/m^2^), infrared images revealed that the car covered with the PF-18-7 membrane was significantly cooler than both the uncovered car and the one covered with cotton. The uncovered car reached a high temperature of 75.8 °C, while the cars covered with cotton and PF-18-7 recorded temperatures of 59.3 and 44.8 °C, respectively. The results suggested that the radiative cooling and waterproof, breathable PVDF-HFP/FAS membranes show strong potential for various practical applications such as automobiles and buildings.

## 3. Materials and Methods

### 3.1. Materials

PVDF-HFP (Mw = 400,000) was purchased from Shanghai Aichun Biotechnology Co., Ltd., Shanghai, China. Dimethylformamide (DMF) was purchased from Jiangmen Jianghai Deshi Instrument Chemical Co., Ltd., Jiangmen, China. FAS-17 (density = 1.5373 g/mL) was supplied by Zhongshan Dishin Chemical Co., Ltd., Zhongshan, China.

### 3.2. Construction of Functional Materials

PVDF-HFP solutions with different concentrations of 12, 14, 16, and 18 wt% were prepared by dissolving PVDF-HFP into DMF. The PVDF-HFP spinning solution was injected into five syringes, which are fixed side by side on a stand with a working distance of 25 cm from the tip to release the paper. The temperature was 23–26 °C, the humidity was 90–95%, the extrusion rate was 2 mL h^−1^, and the applied voltage was 30 kV. The prepared fibrous membranes were denoted as PH-12, PH-14, PH-16, and PH-18, respectively. PVDF-HFP/FAS solutions were prepared by dissolving 18 wt% of PVDF-HFP and 3, 7, 11, and 15 wt% of FAS in DMF. The spinning conditions were the same with PVDF-HFP membranes. The resulting fiber membranes were named PF-18-3, PF-18-7, PF-18-11, and PF-18-15, respectively.

### 3.3. Characterizations

The chemistry structure was determined by Nicolet IS50 Fourier transform infrared spectrometer (FTIR, Thermo Fisher Scientific, USA). The microstructure of the prepared fibrous materials was studied by Scanning electron microscope scanning electron microscopy (SEM, Zeiss Gemini Sigma 300VP SEM, Carl Zeiss Co., Ltd., Jena, Germany). Thickness was measured through a thickness gauge (CHY-C2, Shanghai Precision Instruments Co., Ltd., Shanghai, China). Pore characteristics were studied using a capillary flow porometer (CFP-1100AI, Porous Materials Inc., Ithaca, NY, USA). The porosities of the samples were estimated using the same method as before [16].

### 3.4. Measurements

The air permeability was tested through the YG461E-III automatic air permeability meter (Ningbo Textile Instrument Factory, Ningbo, China). YG825E digital fabric water permeability tester (Ningbo Textile Instrument Factory, Ningbo, China) was employed to characterize water resistance. By using the YG216-II water vapor permeability tester (Wenzhou Darong Textile Instrument Co., Ltd., Wenzhou, China), the WVT rate of fabrics was measured by the Reverse Cup method, and the result was calculated as before [15]. The mechanical properties of fiber membranes were studied using an electronic universal material testing machine (LEGEND2366, Instron Corporation, USA) at the speed of 5 mm min^−1^. The fiber membrane was made into a small sample with a length of 50 mm and a width of 20 mm. The reflectivities of the samples were tested by a UV-Vis-NIR photometer (UV-3600Plus, SHIMADZU, Japan).

## 4. Conclusions

In this paper, waterproof, breathable, and radiative cooling PVDF-HFP/FAS membranes were successfully prepared by a facile one-step electrospinning process. PVDF-HFP was selected as the fibers matrix due to its excellent hydrophobicity, high infrared emissivity, and favorable mechanical properties. FAS was incorporated into the electrospinning solution to further enhance hydrophobicity and solar reflectance. The PF-18-7 composite membrane, composed of 18 wt% PVDF-HFP and 7 wt% FAS, displayed exceptional integrated performance, including water resistance of 62.0 kPa, moisture permeability of 12.4 kg m^−2^ d^−1^, air permeability of 13.3 mm s^−1^, solar reflectivity of 92%, and infrared emissivity of 97.2%. These multifunctional properties demonstrate the potential for large-scale applications in fields such as personal thermal management, transportation, and architecture.

## Data Availability

The data used to support the findings of this study can be made available by the corresponding authors upon request.

## Supplementary Materials

The following supporting information can be downloaded at: https://www.mdpi.com/article/10.3390/molecules30040763/s1, Figure S1: the viscosity of different pure PVDF-HFP fibrous membranes; Figure S2: the average reflectance of different pure PVDF-HFP fibrous membranes; Figure S3: the diameter distributions of different PVDF-HFP/FAS composite fibrous membranes; Figure S4: the average reflectance of different PVDF-HFP/FAS composite fibrous membranes; Table S1: XPS results of PVDF-HFP/FAS membranes.
