# Supplementary material for: One-Step Fabrication of Poly(vinylidene Fluoride-Co-Hexafluoropropylene)/Perfluorodecyltriethoxysilane Fibrous Membranes with Waterproof, Breathable, and Radiative Cooling Properties
Source: Molecules. 2025 Feb 7;30(4):763. doi: 10.3390/molecules30040763 (PMC11858151; doi:10.3390/molecules30040763)
Supplement: Supplementary file 1 [file molecules-30-00763-s001.zip › molecules-3427843-supplementary.pdf]

Supporting Information for

One-step fabrication of poly (vinylidene fluoride-co-hexafluoropropylene)/perfluorodecyltriethoxysilane fibrous membranes with waterproof, breathable and radiative cooling propertiess

Aohan Hou[1], Juan Xie[1], Xiaohui Wu[2], Guichun Lin[1], Yayi Yuan[1], Xi Liu[1], Yancheng Wu[1], Feng Gan[1], Yangling Li[1], Yuxiao Wu[1], Gang Huang[1], Zhengrong Li[1,*], Jing Zhao[1,*]

[1] *College of Textile Science and Engineering, Wuyi University, Jiangmen 529020, China.*

[2] *School of Textile and Garment, Anhui Polytechnic University, Wuhu, Anhui 241000, China.*

* *Correspondence authors:*

Jing Zhao, E-mail address: jingzhaoedu@126.com; Zhengrong Li, E-mail address: lizhengrongwu@163.com.

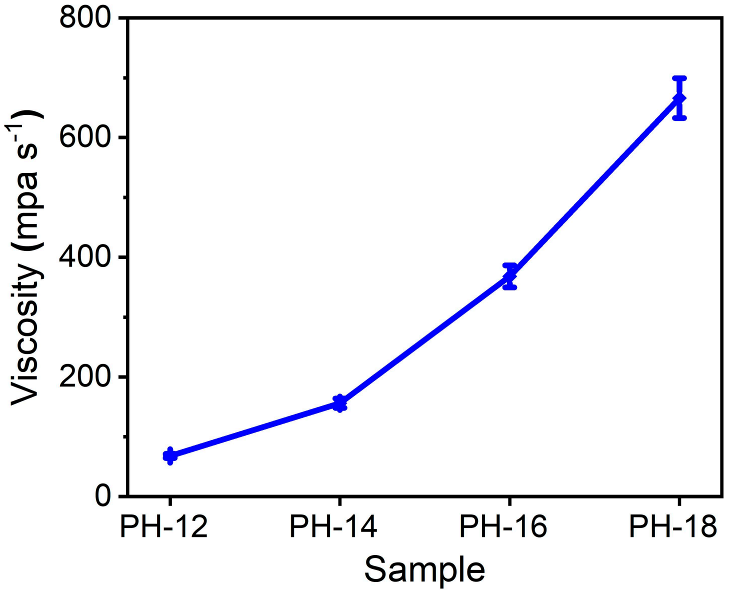

Figure S1. The viscosity of different pure PVDF-HFP fibrous membranes.

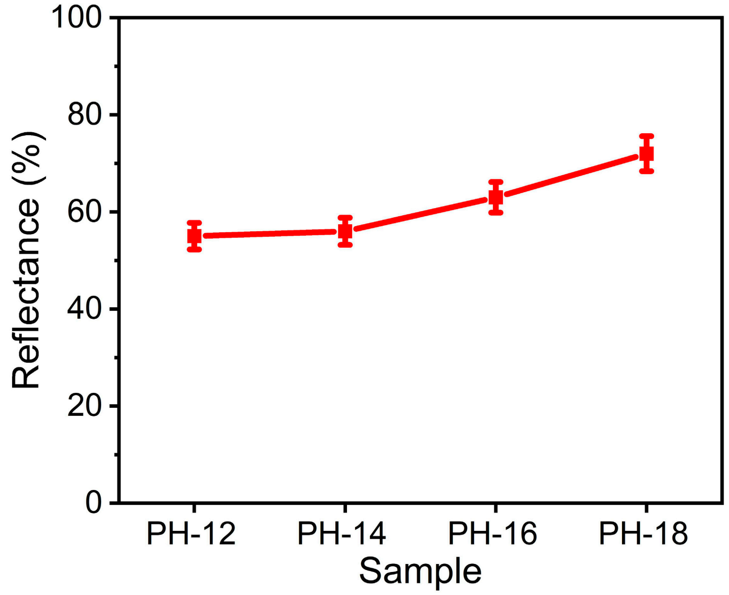

Figure S2. The average reflectance of different pure PVDF-HFP fibrous membranes.

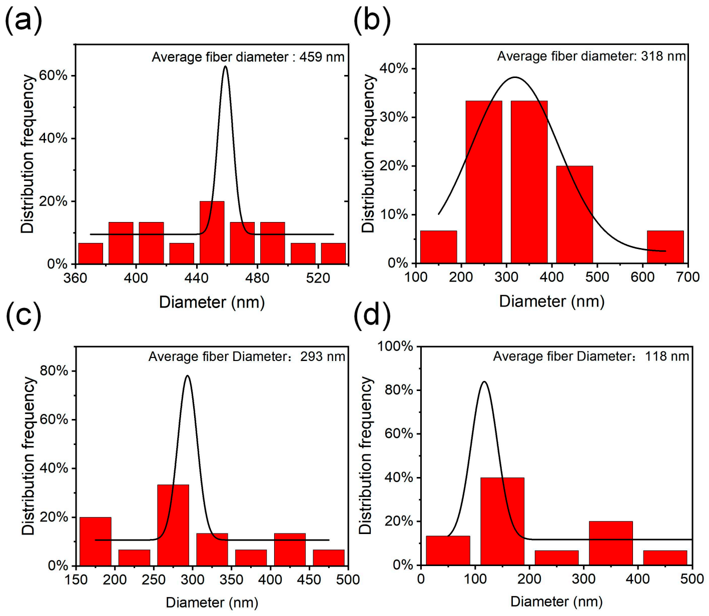

Figure S3. The diameter distributions of different PVDF-HFP/FAS composite fibrous membranes.

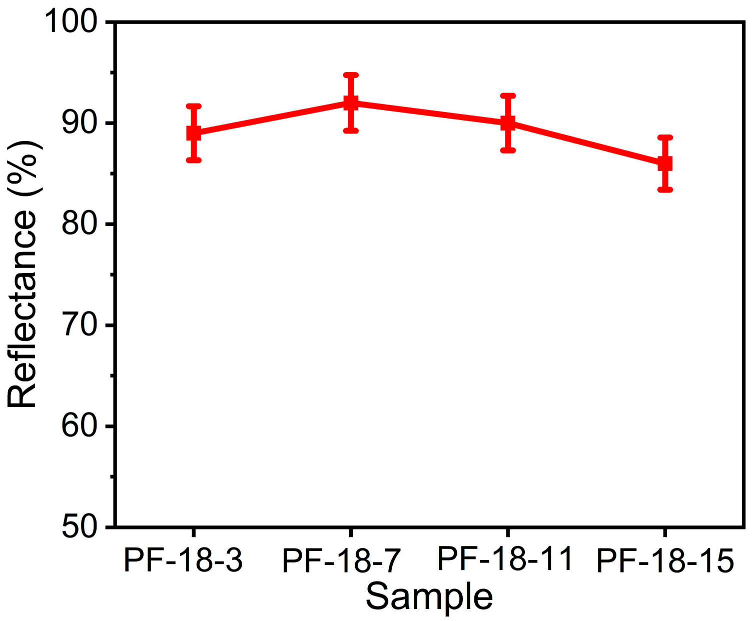

Figure S4. The average reflectance of of different PVDF-HFP/FAS composite fibrous membranes.

Table S1.XPS results of PVDF-HFP/FAS membranes.

Sample	C%	O%	Si%	F%
PF-18-3	43.86	1.98	1.35	52.81
PF-18-7	44.74	2.24	1.42	51.59
PF-18-11	45.95	2.81	1.84	49.41
PF-18-15	44.17	1.48	0.72	53.63
